# Multi-Step Usage of *in Vivo* Models During Rational Drug Design and Discovery

**DOI:** 10.3390/ijms12042262

**Published:** 2011-04-01

**Authors:** Charles H. Williams, Charles C. Hong

**Affiliations:** 1 Division of Cardiovascular Medicine, Department of Medicine, Vanderbilt University School of Medicine, Nashville, TN 37232, USA; E-Mail: Charles.h.williams@vanderbilt.edu; 2 Research Medicine, Veterans Affairs TVHS, Nashville, TN 37212, USA

**Keywords:** phenotypic screen, drug discovery, small molecules, drug design chemical genetics, model organisms

## Abstract

In this article we propose a systematic development method for rational drug design while reviewing paradigms in industry, emerging techniques and technologies in the field. Although the process of drug development today has been accelerated by emergence of computational methodologies, it is a herculean challenge requiring exorbitant resources; and often fails to yield clinically viable results. The current paradigm of target based drug design is often misguided and tends to yield compounds that have poor absorption, distribution, metabolism, and excretion, toxicology (ADMET) properties. Therefore, an *in vivo* organism based approach allowing for a multidisciplinary inquiry into potent and selective molecules is an excellent place to begin rational drug design. We will review how organisms like the zebrafish and *Caenorhabditis elegans* can not only be starting points, but can be used at various steps of the drug development process from target identification to pre-clinical trial models. This systems biology based approach paired with the power of computational biology; genetics and developmental biology provide a methodological framework to avoid the pitfalls of traditional target based drug design.

## 1. Introduction

**Figure 1.**
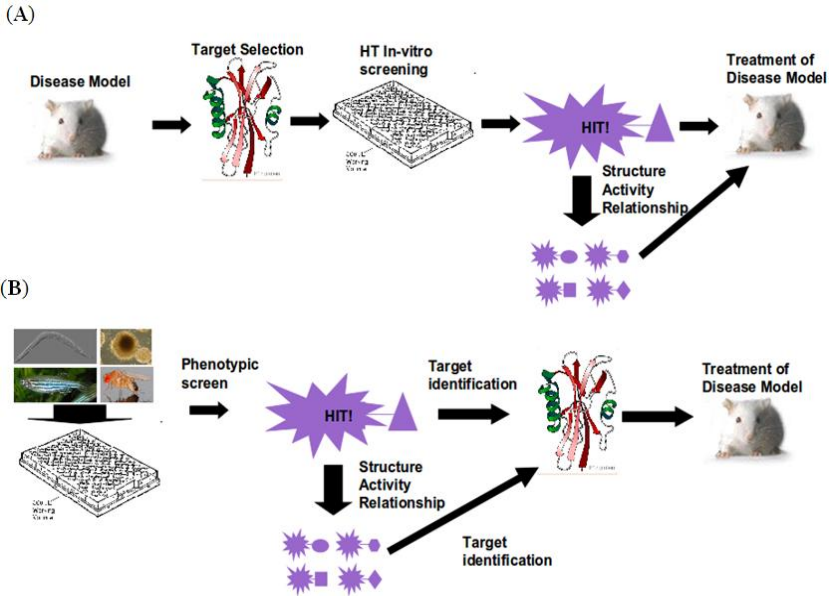
Workflow for two paradigms of drug discovery. (**A**) Conventional “Target centered” drug discovery; (**B**) *In vivo* model based drug discovery.

Cancer, Alzheimer, diabetes; all are leading causes of death in the US. Unlike exogenous factors like HIV/AIDS and influenza, they are the result of endogenous developmental programming behaving in an aberrant fashion. As the average lifespan of human increases, certain biological machineries in our bodies start to break down. Even if all exogenous infections were obliterated from the face of the globe we would still face these diseases. Pharmaceutical companies face the challenge of modulating these developmental processes with small molecules. The human genome consists of approximately 25,000 genes [1]. Of these genes only 3000 of which are thought to be druggable and 50% of those thought to be disease causing [2]. A list of ∼1500 potentially druggable disease causing biomolecules is now the center of focus in the pharmaceutical industry. The current paradigm of drug design revolves around these biomolecule targets and designing and identifying small molecules that modulate the activity of them *in vitro* or *in silico;* this is called a “target centered” approach (Figure 1a). Let us look at the track record of this approach. Only 1 of 5000 discoveries makes it to market from the bench side. The average time it takes a drug to reach the bedside from discovery is 12 years, and a single pharmaceutical agent costs from 500 million to 2 billion dollars to bring to market [3]. How could all but one of 5000 discoveries end up being useless as a therapeutic? The answer may lie in the “target centered” paradigm that has driven drug design for the past 50 years.

This paradigm is not without its successes. The anti-hypertensive agent Captopril produced by Bristol Meyer-Squibb is a potent and reversible inhibitor of Angiotensin-converting enzyme. Approved by the FDA in 1981 it rapidly became an effective treatment for hypertension and heart failure [4]. And Novartis produced an FDA approved drug Aliskiren, in 2007 with a combination of X-ray crystallography and computer aided design [5]. However, most of these target centric designs often fail to meet the standard when ADMET (absorption, digestion, metabolism and toxicity) is evaluated after years of research and millions of dollars. In place of this paradigm, a systems biology approach is emerging using a phenotypic screen that inherently takes into account certain ADMET properties. The following article will present a systematized development method for rational drug design based on phenotype driven discovery.

## 2. Workflow

The first step in *in vivo* phenotypic discovery is the defining the desired phenotype of a “hit” compound. Currently, there are two major types of phenotypic screens. First is a forward chemical genetic screen, which consists of inducing a desired phenotype in a wild typesetting in your model organism (Figure 1B). The second is a therapeutic screen, taking a disease model and reversing it with a compound. However, before either screen can be done a decision must be made about which model system to use. There is also an emerging third type of screen, known as a pathway screen. This screen looks for a change in a particular signaling pathway *in vivo.* The second decision that must be made is which model will be used for the screen. The model organisms are, namely, *Drosophila*, *C. elegans*, zebrafish, or stem cells, which are all discussed below and summarized (Table 1). Further, strategies to identify the molecular target of the hit ligand must be considered; since a compound with completely unknown mechanism of action is unlikely to gain ready acceptance. Various techniques for target identification exist and are not mutually exclusive. Broadly, they are transcriptome profiling, affinity pull down, affinity response target screening, and yeast 3 hybrid screening. At this juncture, it is important to consider whether a “hit” compound is bioactive in live animals, depending on what model was used for initial screening. Assuming the ultimate goal of a chemical screen is to discover novel therapeutics for humans, it would be important to test whether the small molecule intervention robustly elicits desired effects without toxicities in inexpensive model organisms, prior to advancing the small molecule toward much more expensive clinical testing. Indeed, it has been estimated that just 10% improvements in predicting failures before clinical trials could save $100 million per drug [6].

## 3. Organismal Models

Invertebrates are small, low cost and tend to have high fecundity; as such, they are one possible avenue for screening models. One such invertebrate worming its way into drug discovery is *C. elegans*; it is a nematode with a short life cycle (approximately 3.5 days) and can be raised in liquid media while consuming *E. coli*. Each adult hermaphrodite consists of 959 cells and can produce ∼300 larvae by self-fertilization. Since it started being used by Sydney Brenner in 1960s [7], it has been used to study cancer [8], neuronal cell death [9], and cilia [10]. *C. elegans* has also been established as a disease model of Alzheimer’s disease [11], Parkinson’s disease [12], Friedrich ataxia [13], and diabetes mellitus [14]. Recently the small nematode has made progress as a screening tool, in part due to a HTS method of arraying larvae [15]. For example, Kwok and colleagues identified a novel calcium antagonist that targets egl-19, the l-type calcium channel alpha1-subunit [16]. Additionally, an automated image analysis based high-throughput screen utilizing transgenic worms identified known autophagy enhancers that could be used to treat human liver diseases caused by protein misfolding [17]. Despite the versatility of *C. elegans* as a screening model, it has a few important drawbacks. First, its homology to the human genome is relatively low, with approximately 50% of human genes having orthologues [18]. Many organs in the human body do not have corresponding structures in *C. elegans*. The *C. elegans* body is also covered by a thick cuticle that is hard to penetrate [19]. Many compounds will not penetrate it unless a special solvent containing naphthalene and para-dichlorobenzene is added to the media [20]. Overall, *C. elegans* is promising model for *in vivo* small molecule screening to yield tools for simple biologically conserved pathways.

Another invertebrate that shows potential for small molecule screening is the fruit fly *Drosophila melanogaster*. It has been used for over a century in genetics research. *Drosophila* is anatomically more similar to mammals and has more than 60% genomic homology to humans [21]. As with *C. elegans* there are many mutants and even a fair number of disease models ranging from muscular dystrophy [22], to Alzheimers [23]. Recently this model has successfully been used by Chang and colleagues in a therapeutic screen. Briefly, a screen of 2000 compounds resulted in identification of nine molecules that rescue the *Drosophila* model of fragile X syndrome [24]. This screen, in addition to providing new small molecule tools, uncovered a novel function for muscarinic cholinergic receptors acting in parallel to the GABAergic pathway in rescuing fragile X syndrome phenotypes. Despite these impressive results, the drawbacks of using *Drosophila* as a small molecule screening model are three-fold. First, the organism is covered with a cuticle that is hard to penetrate. Secondly, *Drosophila* does not grow in a liquid media so precise dosing of small molecules in a high throughput manner is difficult. Thirdly, as with *C. elegans*, the *Drosophila* model lacks some anatomical (e.g., closed circulatory system) and genetic components that are present in humans.

As a vertebrate model of human disease, mouse has long been the gold standard. However, the size, labor and time requirements for mice make them cost prohibitive for conducting small molecule screens. However, there has been some limited success in chemical screening using mice. A proof of principle comparison of anti-TB drugs has been conducted and shows promise as a methodology for conduction large scale screens for TB therapeutics [25]. In recent years, another vertebrate model has emerged at the forefront of small molecule screening. Over the past 20 years the zebrafish has made a substantial impact in biological research. It has been used to study multiple areas ranging from vascular development [26,27] and neural development [28], to disease models for cancer [29] and melanocyte development [30]. The embryos are roughly 1 mm in size and 3–6 embryos can comfortably be arrayed in the wells of a 96-well plate [31]. Its size, low cost, and fecundity make zebrafish an attractive model for basic research. In addition, the liquid media, genetic homology to humans (over 70%), and rapid development of most organs within 48 h post fertilization, make it an ideal model for small molecule screening. With these advantages, the small teleost is swimming its way into the field of chemical genetics. There are four major models for phenotypic screening in zebrafish; they are chemical genetic, therapeutic, transgene assisted and pathway based screens (Table 2). We have, in a chemical genetic screen, used perturbation of dorso-ventral (front to back) polarity in zebrafish embryos to discover dorsomorphin, the first selective Bone morphogenic protein (BMP) type I receptor [32]. Dorso-ventral patterning in zebrafish is established primarily through a functional antagonism between Wnt and BMP, but perturbations in numerous other pathways can distort the overall embryonic patterning. In a slightly more focused, transgene assisted, approach screening for anti-angiogenic agents, transgenic fish expressing GFP under a vasculature specific promoter were used in an automated and quantitative screen. This identified two known anti-angiogenic agents along with one other novel compound [33]. Zebrafish have also been used to screen for modifiers of specific pathways such as Fgf through the use of a florescent reporter [34].

In addition to organism based phenotypic screens, chemical screen using pluripotent stem cells has emerged as a viable alternative. A major benefit of screening in human derived stem cells is that one doesn’t need to worry about translatability in terms of conservation and orthology. Stem cells are grown in liquid media and can be arrayed in a 96-well format to form uniform sized embryoid bodies [35]. Stem cells can give rise to all three germ layers and any cell type should be derived. Small molecules have successfully been used to create numerous cell types including cardiomyocytes [36,37]. Because of this attractive therapeutic potential, for directed differentiation, a small molecule screens using stem cells (R1 cells) was conducted by Zhu and colleagues for small molecules that promoted differentiation into definitive endoderm by assaying for expression of a sox17-rfp [38]. Additionally, ectoderm derived neurospheres were also used for assay for neurogenic compounds in a phenotypic screen [39]. However, as with many cell based assays this model for phenotypic screening could also potentially lead to identification of compounds that have poor ADME properties. This methodology could be applied to the generation of any number of cell types for which specific markers are available, such as Beta-cells and could substantially help the field of regenerative medicine.

## 4. Target Identification

Traditional drug design starts with the identification of a possible therapeutic target. For phenotypic screens target identification is the bottle neck for drug development. However, major advances are being made in the field for more efficient and rapid identification. These techniques can for our purposes be broken into two broad categories; techniques requiring modification of the small molecule and techniques that can use the native molecule.

The first two methods, that don’t require modification of the ligand, are based on comparative transcriptome profiling. By utilizing a network systems biology approach one can identify the central nodes of affected gene clusters. This can yield a broad view of the mechanism of action [40]. One can also use hierarchical clustering which has been successful in both yeast and rat tissue [41]. This however provides documentable cell-type specific effects, and also microarray specific effects. An alternative approach is through the use of the Connectivity Map [42]. This takes a rank based pattern matching strategy applied to a database of over 7000 expression profiles representing 1309 compounds to identify similarities and thus potential target pathways [43].The problem with the listed techniques thus far, has been that they are unable to pinpoint specific binding partners. There is a new technique called Drug Affinity Response Target Screening (DARTS) [44]. When a small molecule binds a molecule there is a physical response. This response can be proteolytically protective, and the resultant protected peptide sequence can yield the target protein. The efficacy of this method has been tested with both high and low binding affinity molecules. This technique does not depend on an *in vivo* response and gives large amount of flexibility.

If one has a good understanding of the structure activity relationship for the compound and the phenotype one can alter the small molecule so it can be affixed to a linker. Once a linker is affixed to the small molecule, there are two approaches that can be taken. The first is the chemical proteomic approach; this approach is based on affinity chromatography and utilizes the pull down of candidate proteins. This has been the golden standard in the field for many years. With advances in MS technology and protein technologies there are new flavors of this approach that use isotope labeled proteins (a significant review of these methods was written by Rix and Superti-Furga [45]). Another technique that can be used is a variation of the yeast two hybrid screen. The yeast 3 hybrid screen requires the small molecule to be linked to a methotrexate moiety (the anchor). This molecule will attach itself to the hybrid dihydrofolate reductase-LexA DNA binding domain via the methotrexate. The small molecule bait will bring any target molecules (from a cDNA library of Gal4 activation domain fusions) within range of the DNA binding domain to elicit a His-3 reporter [46]. This is a versatile system and the cDNA libraries are commercially available from a number of sources for a number of different model systems.

## 5. SAR and Compound Optimization

Most SAR studies are done with *a priori* knowledge of the target molecule or pathway. Recent advances in computer technology have made molecular modeling based on crystallized protein structure feasible for even small laboratories [47]. Traditionally, an *in vitro* assay is used for testing analogs to determine SAR. However, it is possible to conduct SAR *in vivo* using a phenotypic model as a read out. Hao and colleagues used the zebrafish model to perform the first *in vivo* SAR of dorsomorphin analogues. With this system they were able to derive compounds with differing specificities for Vegf and Bmp receptors [48]. Although the target for dorsomorphin was known, this new paradigm of *in vivo* SAR could be used prior to any knowledge of target pathways as it only requires a simple read-out. This *in vivo* approach also helps in identifying not just the most potent forms of the compound; but also takes into account the *in vivo* permeability of the compound.

## 6. Vertebrate Toxicity

**Figure 2.**
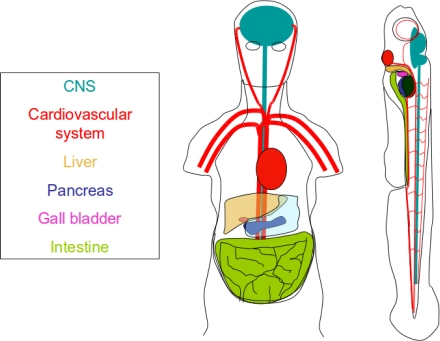
Conservation of organ systems between zebrafish and humans. The zebrafish is a versatile model that is useful not only drug discovery but rapid development of organ systems makes it ideal for assessing biochemical safety and toxicity

One of the major reasons potential drugs fail before they reach the market is because of the off target effects that manifest during clinical trials. The International Conference on Harmonization (ICH) S7A guidelines state that prior to clinical trial in humans, that pharmacophores must be evaluate on the vital functions, namely the circulatory system, CNS, G.I., and skeletal systems [49]. Of the models discussed above, only the zebrafish and mouse have all these systems (Figure 2). Traditionally, toxicological data is obtained from mouse. This is however, as mentioned before, a costly model system. In recent years zebrafish have emerged as a viable, low cost alternative for determining compound affects on these major organ systems and could be a useful tool for identifying liabilities of pharmacophores during early stages of drug development.

The zebrafish circulatory system forms and is functional by 23 h post fertilization (hpf), and by 48 hpf the heart has undergone looping to form a distinct atrium and ventricle. The transparency of the zebrafish embryo allows for easy visualization of both the heart and blood circulation. A screen was done with 23 drugs known to cause QT prolongation and *torsades de pointes* in man, to identify if zebrafish would phenocopy the results in 3 days post fertilization (dpf) zebrafish embryos. The results identified 18 that caused brachycardia [50]. Furthermore, a transgenic line that expresses GFP under a cardiac specific promoter has been developed and an automated method of determining heart rate in a high throughput manner has been developed [51]. For further cardiovascular effects, zebrafish blood can be visualized for hemorrhages and an image capture analysis of single erythrocytes can be conducted to assess the contractility of the heart [52]. Within the first 48 h post fertilization zebrafish develop a touch response, at 68 hpf they have a visual startle response, are capable of free swimming at 96 hpf, and develop an auditory startle response by 5 dpf. Screens have been conducted on all of these nervous system responses and have shown remarkable predictability of identifying problems also caused in humans [53–56]. For example, in a study of 8 compounds that can cause visual impairment in man, 6 inhibited the optokinetic motor response correctly in zebrafish [57]. At 36 hpf the zebrafish digestive tract begins to form, and it becomes fully functional and zebrafish are fed exogenously by 5 dpf [58]. As with blood cells, the optical clarity of the zebrafish allows for easy visualization of the peristaltic contractions of the intestine. In a study of compound effects on gut mobility 8 of 10 compounds showed corresponding decreased contractility in zebrafish [58]. Finally, as a vertebrate the zebrafish model allows one to assess bone mineralization by 10 dpf. This is a proof of principle as the study was conducted with prednisolone which substantially decreased bone mineralization in zebrafish [59]. The flexibility and anatomical conservation between man and zebrafish make it ideal for assessing toxicity of various organ systems.

## 7. Afterword

Rational drug design has traditionally depended on a thorough understanding of the disease to be treated so a target protein can be selected and ligands can be screened against that molecular target. However, many diseases are complex, and regardless of the progress made in understanding the disease a coherent model cannot be established. This is particularly true for psychological and neurological diseases such as Alzheimer’s and Parkinson’s disease. In the treatment of many psychological disorders the only pharmaceuticals available target serotonin, dopamine, or norepinephrine signaling. These diseases are diagnosed based on behavior; therefore a model with quantifiable behaviors is necessary for *in vivo* screening. Recently, a screen for chemical modulators of wake/rest cycle was conducted using zebrafish [60]. This study implicated a novel pathway involving ERG (ether-a-go-go) potassium channel proteins. Another potential avenue for *in vivo* screening is cancer. Zebrafish can be injected with human cancer cells at 2 dpf and assessed for both angiogenic response (1 day post implantation) and metastatic behavior (5 days post implantation) [61]. Conducting a small molecule screen on these fish could yield novel therapeutics that target the cancer cells specifically in an *in vivo* environment. Furthermore different cancer cell lines respond differently and as such this screen could be conducted on different lines of cancer cells and yield different results. Whether a disease is well understood or not, phenotypic screens using *in vivo* small animal models like the zebrafish show great promise for aiding drug discovery and development at many steps of the drug development process.

## Figures and Tables

**Table 1.** Comparison of *in vivo* small molecule discovery models.

	***C. elegans***	***D. melanogaster***	***D. rerio***	***M. musculus***
**Generation Time**	3–5 days	10–14 days	3–4 months	6–8 weeks
**Media**	Solid or liquid	Solid	Liquid	N/A
**Ease of Obtaining Embryos**	+++++	+++++	++++	N/A
**Number of Genes**	∼19,000	∼13,000	∼25,000	∼25,000
**Homology to Human Genome**	>50%	>60%	>70%	>90%
**Annual Cost**	+	+	++	++++

**Table 2.** Summary of types of phenotypic screens in zebrafish.

**Embryo**	**Type of Screen**	**Readout**
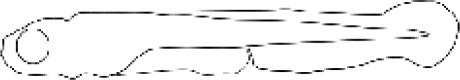 Wild type	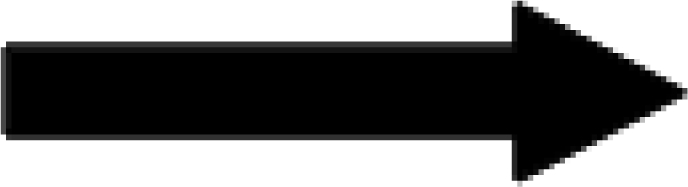 Chemical genetic	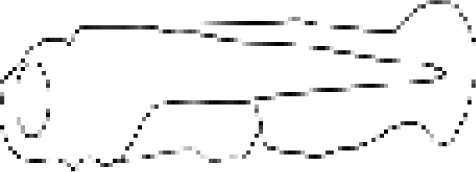 Morphological defect
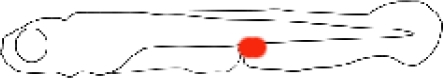 Disease model (eg. Blood pooling)	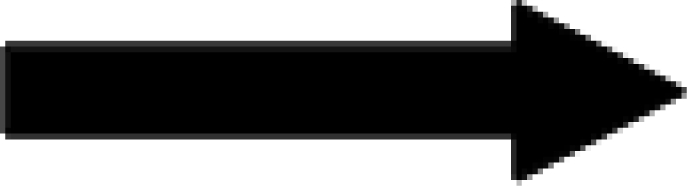 Therapeutic	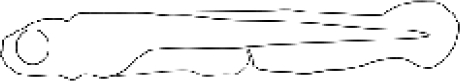 Restored to wild type
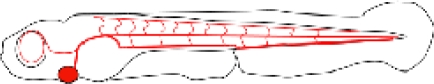 Trangenic Embryo Tg(Flk:dsRed)	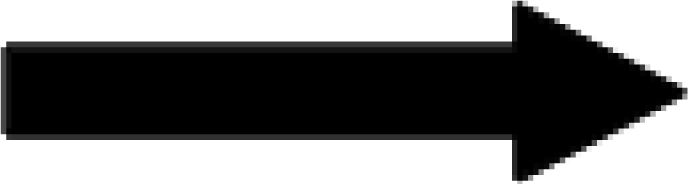 Transgene assisted	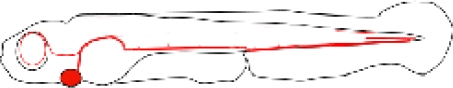 Altered anatomy visualized through transgenic marker
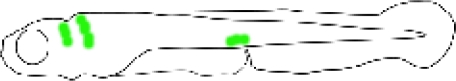 Transgenic Reporter Line Tg(Top:dGFP)	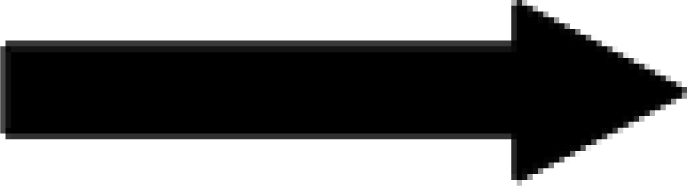 Pathway Reporter inhibitor /enhancer	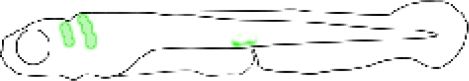 Down regulated Reporter gene

**Table 3.** Overview of molecular target identification technologies.

**Strategy**	**Advantages**	**Disadvantages**
**Affinity**	Traditionally used and readily accepted	Requires sophisticated equipment
**Chromatography**		Low throughput
		Requires chemical modification
**Expression Profiling**	No chemical modifications	Data can be noisy
	High throughput	Requires sophisticated bioinformatics
		Imprecise
**Yeast 3 Hybrid**	High throughput	Non native environment
		Not suitable for membrane bound proteins
		Requires chemical modifications
**DARTS**	No chemical modifications	Low throughput
	Does not require high affinity	
